# Detection and Characterization of Carbapenemases in *Enterobacterales* With a New Rapid and Simplified Carbapenemase Detection Method Called rsCDM

**DOI:** 10.3389/fmicb.2022.860288

**Published:** 2022-04-28

**Authors:** Quanfeng Liao, Yu Yuan, Weili Zhang, Jin Deng, Siying Wu, Ya Liu, Yuling Xiao, Mei Kang

**Affiliations:** Department of Laboratory Medicine, West China Hospital of Sichuan University, Chengdu, China

**Keywords:** phenotypic assay, carbapenem-resistant *Enterobacterales*, carbapenemase, AmpC, rsCDM

## Abstract

**Objective:**

This study aimed to develop a new rapid and simplified carbapenemase detection method (rsCDM) for detection and characterization of carbapenemase with 3-aminophenylboronic acid (APBA), ethylenediaminetetraacetic acid (EDTA), and cloxacillin (CLO) β-lactamase inhibitors.

**Methods:**

A panel of 182 carbapenem-resistant *Enterobacterales* (CRE) strains with *blaKPC* (88), *blaNDM* (60), *blaIMP* (10), *blaVIM* (3), *blaOXA-181* (5), *blaKPC*, and *blaNDM* (7), porin changes in combination with an extended-spectrum β-lactamase (ESBL) (3) or AmpC hyper-production (6) and 43 carbapenem-susceptible *Enterobacterales* isolates were used to evaluate the performance of rsCDM and EDTA-carbapenem inactivation method (eCIM). Carbapenemase class was determined with specific inhibitors at 4, 6, and 18 h by rsCDM, and the difference between imipenem (IMI) and meropenem (MEM) disks was simultaneously compared.

**Results:**

The sensitivity of rsCDM using IMI was 97.1% at the three time points, with a specificity of 100%, independent of the culture duration. Similar to IPM, MEM disk also showed high sensitivity (97.1%) and specificity (100%) at 6 h. And the sensitivity of eCIM was 95.4% and the specificity was 100%. Based on a decision algorithm, the characterization number of IMI and MEM in KPC-producing isolates was 88 vs. 87, metallo-β-lactamases (MBLs) was 73 vs. 72, KPC and NDM carbapenemase was 7 vs. 7 at 4 h, respectively. After 6 h, the category number changed insignificantly except for isolates with combined AmpC overproduction and porin changes, showing an increase in IMI (6) and MEM (2), and there was no difference in the results between 6 and 18 h for the two tablets. OXA-181-producing strains can’t be distinguished by rsCDM. For eCIM, the characterization number in KPC-, OXA- 181-, and MBLs-producing strains was 88, 5, and 72, but it failed to detect multi-enzyme-producing isolates (KPC and NDM).

**Conclusion:**

rsCDM accurately discriminated carbapenemase within 4 h and could differentiate multi-enzyme (KPC and NDM) and AmpC in conjunction with porin changes strains. Hence, rsCDM represents a rapid, simple, easy readout, and accurate tool that can be used without any specialized equipment.

## Introduction

The emergence and spread of CRE is a major clinical and public health concern. These bacteria cause high mortality and are associated with high treatment costs, requiring a combination of agents (Tilahun et al., 2021). Carbapenem resistance in CRE is principally conferred by carbapenemase production. Additionally, hyper-production of AmpC β-lactamases, extended-spectrum β-lactamases combined with altered membrane permeability and high discharge pump also result in carbapenem resistance (Logan and Weinstein, 2017). Carbapenemase can be classified into three different classes of β-lactamases according to the Ambler classification: KPC, IMI, and GES belong to class A, NDM, IMP, and VIM belong to class B, OXA-48-like belongs to class D (Ambler, 1980; Bush and Fisher, 2011), while AmpC enzyme belongs to class C. Carbapenemase class determination can guide antimicrobial therapy, as new agents (e.g., ceftazidime-avibactam) are active against CRE strains producing class A, OXA-48-like and class C enzyme except for Ambler class B carbapenemase (Tilahun et al., 2021).

Genotypic assays are the gold standard for detecting the carbapenemase genes. However, phenotypic detections are convenient and manageable, including biochemical tests, growth-based assays, immunochromatographic assays and the detection of carbapenem hydrolysis by MALDI-TOF MS (Giske et al., 2011; Papagiannitsis et al., 2015; Silva et al., 2017; Caméléna et al., 2018; Jing et al., 2018, 2019; CLSI, 2020; Petit et al., 2020). Among these phenotypic assays, only a few tests can determine the class of carbapenemase (Giske et al., 2011; Silva et al., 2017; CLSI, 2020; Petit et al., 2020). eCIM recommended by CLSI (2020) can distinguish carbapenemase, but it requires 18–24 h and its steps are cumbersome. Additionally, eCIM can’t detect multi-enzyme (KPC and NDM) and AmpC β-lactamases. In 2010, Giske et al. (2011) reported a method to detect carbapenemase and AmpC enzymes in *Klebsiella pneumoniae* using meropenem disks supplemented with 3-aminophenylboronic acid (APBA), dipicolinic acid, ethylenediaminetetraacetic acid (EDTA), and cloxacillin (CLO). Silva et al. (2017) published a method of detecting carbapenemase and AmpC enzymes with phenylboronic acid, cloxacillin, and EDTA in 2017. However, these methods (Silva et al., 2017; Jing et al., 2018) failed to distinguish multi-enzyme and required 16–18 h. In 2020, a new mCIMplus method (Petit et al., 2020) could detect carbapenemase activity within 8 h, but it took 20 h to characterize the Ambler classification.

In the present study, we developed a new, rapid and simplified carbapenemase detection method (rsCDM), using imipenem or meropenem supplemented with three β-lactamase inhibitors (EDTA, APBA, and CLO) to detect and discriminate carbapenemase classes in carbapenemase-producing *Enterobacterales* (CPE), and overproduction of AmpC in combination with porin abnormality in non-carbapenemase-producing carbapenem-resistant *Enterobacterales* (non-CP-CRE) isolates within 4–6 h.

## Materials and Methods

### Bacteria

A total of 225 well-characterized *Enterobacterale*s strains were isolated from the West China Hospital of Sichuan University. The panel consisted of *Klebsiella* spp. (166), *Escherichia coli* (*E. coli*) (39), *Enterbacter hormaechei* (13), *Enterobacter cloacae* (6), and *Citrobacter koseri* (1). Of these, 182 strains were resistant to carbapenem, and 173 expressed the carbapenemase genes *blaKPC-2* (88/173, 50.9%), *blaIMP-4* (7/173, 4.0%), *blaIMP-1* (2/173, 1.2%), *blaIMP-8* (1/173, 0.6%), *blaVIM-1* (3/173, 1.7%), *blaNDM-1* (15/173, 8.7%), *blaNDM-5* (44/173, 25.4%), *blaNDM-7* (1/173, 0.6%), *blaKPC-2* and *blaNDM-5* (3/173, 1.7%), *blaKPC-2* and *blaNDM-1* (4/173, 2.3%), and *blaOXA-181* (5/174, 2.9%) (Supplementary Table 1). Carbapenem-resistant *K. pneumoniae* (CRKP) isolates with ESBLs in combination with abnormalities of *blaOmpK35/blaOmpK36* (3), as well as CRKP strains overproducing AmpC accompanied by *blaOmpK35/blaOmpK36* changes (6) were included. The remaining 35 *K. pneumoniae* and 8 *E. coli* strains were susceptible to carbapenem. All strains were identified at the species level by MALDI-TOF MS (Bruker Daltonics, Bremen, Germany) using a Vitek 2 compact instrument (BioMerieux, Marcy-l’Étoile, France). The whole genomes of 63 isolates from in our laboratory were previously sequenced with Illumina technology, including 6 AmpC- and 3 ESBLs accompanied by *blaOmpK35/blaOmpK36* producing CRE, and remaining isolates were previously characterized by PCR (Liao et al., 2021). Minimum inhibitory concentration (MIC) of imipenem, meropenem, and ertapenem were determined by *E*-test (Autobio, Zhengzhou, China) or broth microdilution, and the results were interpreted by CLSI 2021 breakpoints. *K. pneumoniae* ATCC 700603 and *E. coli ATCC 25922* were used as quality control strains. Further information about carbapenem MICs, β-lactamase genes and results of rsCDM and eCIM is provided in Supplementary Table 1.

### Rapid and Simplified Carbapenemase Detection Method

**FIGURE 1:**
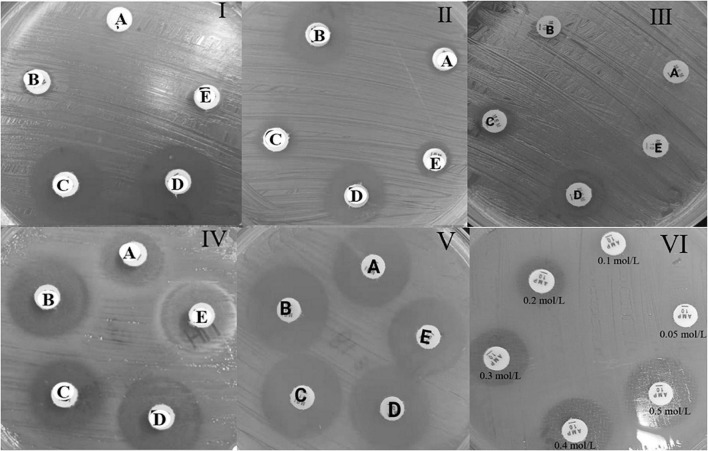
Example of the carbapenemase and AmpC enzyme detection test with reading at 4 h, for the rsCDM test. A, imipenem; B, imipenem + APBA; C, imipenem + EDTA; D, imipenem + APBA + EDTA; E, imipenem + CLO. MBL carbapenemase **(I)**, KPC carbapenemase **(II)**, KPC and MBL carbapenemase **(III)**, AmpC with porin changes **(IV)**, ESBL enzyme **(V)**, EDTA negative control **(VI)**.

**FIGURE 2:**
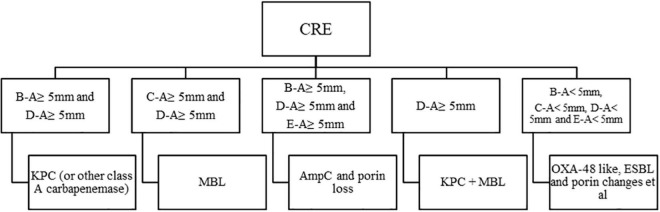
Algorithm for interpretation of results, a 10 μg imipenem disk was used, in the presence or absence of β*-*lactamase inhibitors. APBA, 3-aminophenylboronic acid; CLO, cloxacillin; EDTA, ethylenediaminetetraacetic acid; KPC, KPC carbapenemase; MBL, metallo-β-lactamase; ESBL, extended-spectrum β-lactamase. A, imipenem; B, imipenem + APBA; C, imipenem + EDTA; D, imipenem + APBA + EDTA; E, imipenm + CLO.

rsCDM is a new method to detect and distinguish carbapenemases based on previous methods (Giske et al., 2011; Silva et al., 2017; Liao et al., 2021). Imipenem disks (Autobio, Zhengzhou, China) were placed on MH agar plates, followed by the addition of 10 μL of three different β-lactamase inhibitors: 50 mg/mL APBA (Macklin, Shanghai, China), 0.1 mol/L EDTA (Solarbio, Beijing, China) and 75 mg/mL CLO (Sigma-Aldrich). A 3.0 McFarland inoculum was prepared and spread on MH agar plates (Autobio, Zhengzhou, China). Five 10 μg imipenem disks (named A, B, C, D, E) were placed on the plate: A, imipenem; B, imipenem + APBA; C, imipenem + EDTA; D, imipenem + APBA + EDTA; E, imipenem + CLO. The plates were incubated at 35 ± 2^°^C, and zone diameter was measured at 4, 6, and 18 h. According to previous researches and quality control results (Giske et al., 2011; CLSI, 2020; Liao et al., 2021), an increase of ≥ 5 mm in zone diameter around disks containing β-lactamase inhibitors as compared to disks with imipenem alone, was considered as a positive result for APBA, EDTA, and CLO. Hence, isolates with a subtraction zone ≥ 5 mm for APBA were considered as KPC producers, those with a zone difference ≥ 5 mm only for EDTA were considered likely producers of MBL, and the production of both MBL and KPC carbepenemase was considered for disk D vs. disk A ≥ 5 mm and a mismatch in the criteria of single enzyme, strains with a difference ≥ 5 mm for APBA and CLO were considered possible producers of AmpC. Isolates with zone difference < 5 mm for APBA, CLO, and EDTA-impregnated disks were considered possible producers of another β-lactamase (OXA-48/ESBL) or porin loss (Jing et al., 2019). For the rsCDM test, pinpoint colonies within any zone of inhibition were ignored. The positive and negative quality control (QC) strains used in this study were *Klebsiella pneumoniae* ATCC BAA-1705 (*blaKPC*-positive by PCR), *Klebsiella pneumoniae* ATCC BAA-1706, and *Klebsiella pneumoniae* ATCC BAA-2146 (*blaNDM*-positive by PCR). Since EDTA could show bacteriostatic effect on specific concentrations, we made 6 EDTA concentrations (0.05, 0.1, 0.2, 0.3, 0.4, and 0.5 mol/L) to perform rsCDM with *K. pneumoniae* ATCC 700603, and found that EDTA concentration of negative control inhibited the growth of testing strains with 0.2 mol/L EDTA (Figure 1). The decision algorithm is presented in Figure 2. Examples of carbapenemase/AmpC enzymes detection and its characterization are presented in Figure 1.

At the same time, the testing capability of meropenem was evaluated.

### EDTA-Carbapenem Inactivation Method

eCIM was performed and interpreted as recommended by CLSI (2020).

### Statistical Analysis

Chi-square test was used to examine the difference in the results between rsCDM and the two existing methods (sequencing and eCIM), and the Kappa coefficients were further provided to indicate the degree of consistency. Data analysis was performed with SPSS 22.0. The significance was set at *p* < 0.05.

## Results

The results are displayed in Table 1. Among the 173 tested CPE strains, 168/173 and 166/173 CPE isolates were detected and characterized with imipenem and meropenem at 4 h. When using meropenem, the number increased from 166 to 168 as the incubation period ranged from 4 to 6 h. All CPE strains were correctly classified according to the Ambler system by rsCDM with the exception of three OXA-181-producing *K. pneumoniae* and two OXA-181-producing *Escherichia coli* strains, which showed negative. For 88 isolates carrying class A (KPC) and 73 class B carbapenemases, imipenem correctly identified 88 (100% agreement) and 73 CPEs (100% agreement) at 4 h, respectively, and 87 (98.9% agreement), 72 (98.6% agreement) for meropenem, with detection rate increasing to 100% after 6 h of meropenem incubation. As for class C enzyme, six AmpC-producing isolates were distinguished in 6 h using imipenem, and two strains showed AmpC production by meropenem at 6 h, which was not associated with culture duration. 7 multi-enzyme (KPC and MBL) strains were classified correctly by rsCDM. None of the CPE-negative isolates was classified as positive by rsCDM, and none of the CPE-positive isolates was classified as indeterminate. The sensitivity of rsCDM by imipenem for CPE was 97.1% at 4, 6, and 18 h, 96.0, 97.1, 97.1% for meropenem, respectively, and its specificity was 100%. Compared with rsCDM, eCIM also exhibited great ability to identify KPC- (100%), MBLs- (98.6%), and OXA-181-producing (100%) isolates, but it failed to detect strains producing KPC and NDM carbapenemases.

**TABLE 1:** Performances of the rsCDM and eCIM tests in *Enterobacterales* strains.

β-lactamase (n)		Sensitivity of rsCDM using imipenem (%)			Sensitivity of rsCDM using meropenem (%)			Sensitivity of eCIM (%)
		4 h	6 h	18 h	4 h	6 h	18 h	
Class A	KPC (88)	88 (100%)	88 (100%)	88 (100%)	87 (98.7%)	88 (100%)	88 (100%)	88 (100%)
Class B		73 (100%)	73 (100%)	73 (100%)	72 (98.6%)	73 (100%)	73 (100%)	72 (97.3%)
	NDM (60)	60 (100%)	60 (100%)	60 (100%)	60 (100%)	60 (100%)	60 (100%)	60 (100%)
	IMP (10)	10 (100%)	10 (100%)	10 (100%)	10 (100%)	10 (100%)	10 (100%)	9 (90%)
	VIM (3)	3 (100%)	3 (100%)	3 (100%)	2 (66.7%)	3 (100%)	3 (100%)	3 (100%)
Class D	OXA-181 (5)	0	0	0	0	0	0	5 (100%)
Class A + B	KPC and NDM (7)	7 (100%)	7 (100%)	7 (100%)	7 (100%)	7 (100%)	7 (100%)	0
All carbapenemases (173)		168 (97.1%)	168 (97.1%)	168 (97.1%)	166 (96.0%)	168 (97.1%)	168 (97.1%)	165 (95.4%)
Class C	AmpC (6)	0	6 (100%)	6 (100%)	0	2 (33.3%)	2 (33.3%)	0
	None (46)	0	0	0	0	0	0	0

The zones of diameter were read at 4, 6, and 18 h to explore the correlation between the results and incubation period. No change of characterization number was found at two time points (6 and 18 h), but subtraction inhibition diameters of 18 h were bigger than 6 h, which made it easier to interpret the results in clinical practice (Table 2).

**TABLE 2:** The rsCDM subtraction zone diameter with imipenem and meropenem.

		Subtraction zone diameter with IMI at 4 h				Subtraction zone diameter with IMI at 6 h				Subtraction zone diameter with IMI at 18 h				Subtraction zone diameter with MEM at 4 h				Subtraction zone diameter with MEM at 6 h				Subtraction zone diameter with MEM at 18 h			
Type of carba penemase	Genes	B vs. A (mm)	C vs. A (mm)	D vs. A (mm)	E vs. A (mm)	B vs. A (mm)	C vs. A (mm)	D vs. A (mm)	E vs. A (mm)	B vs. A (mm)	C vs. A (mm)	D vs. A (mm)	E vs. A (mm)	B vs. A (mm)	C vs. A (mm)	D vs. A (mm)	E vs. A (mm)	B vs. A (mm)	C vs. A (mm)	D vs. A (mm)	E vs. A (mm)	B vs. A (mm)	C vs. A (mm)	D vs. A (mm)	E vs. A (mm)
Serine carba penemase	*blaKPC* (88)	5–10	0–4	5–11	0-4	5–11	0–3	5–11	0–4	5–12	0–4	5–11	0–2	5–9	0–4	5–10	0–1	5–10	0–4	5–10	0–2	5–11	0–1	5–11	0–1
MBLs (73)	*blaNDM* (60)	0–3	5–12	5–13	0-4	0–3	5–16	5–16	0–3	0–4	8–17	7–16	0–4	0–3	5–13	5–15	0–4	0–3	5–13	5–13	0–4	0–2	5–15	5–17	0–2
	*blaIMP* (10)	0–1	5–9	5–9	0-3	0–1	5–9	5–8	0–1	0–1	5–11	5–11	0–3	0	5–9	5–9	0–2	0–1	5–9	5–10	0–2	0–3	5–13	5–14	0–2
	*blaVIM* (3)	0–1	5–8	6–8	0-1	0–1	7–9	7–9	0	0–1	5–10	6–11	0–2	0	3–9	3–9	0	0	6–9	6–9	0	0–1	8–11	5–11	0
	All (73)	0–3	5–12	5–13	0-4	0–3	5–16	5–16	0–3	0–4	5–17	5–16	0–4	0–3	3–13	3–15	0–4	0–3	5–13	5–13	0–4	0–3	5–15	5–17	0–2
Multi-enzyme (7)	*blaKPC* and *blaNDM*(7)	0–4	0–4	8–12	0	0	0–3	5–10	0–2	0	0	6–9	0	0	0	7–9	0	0	0–2	7–10	0	0	0	6–9	0
AmpC (6)		6–8	0–1	0–3	5-8	5–7	0–2	0–3	5–7	5–7	0–3	6–7	5–6	5–7	0–2	1–2	5–8	1–7	0–1	0–6	0–7	4–5	0–4	5–6	3–5

*IMI, imipenem; MEM, meropenem.*

The difference between rsCDM, sequencing and eCIM results was examined by the chi-square test. For 225 *Enterobacterales* strains, there was no statistical difference between rsCDM, sequencing and eCIM (Table 3).

**TABLE 3:** The statistic results of sequencing, rsCDM and eCIM for 225 *Enterobacterales* isolates.

Methods	χ^2^	*P-*value	Kappa
Sequencing vs. rsCDM using IMI at 4 h	1.523	0.217	0.86
Sequencing vs. rsCDM using IMI at 6 h	0.329	0.567	0.94
eCIM vs. rsCDM using IMI at 4 h	0.104	0.747	0.84
eCIM vs. rsCDM using IMI m at 6 h	0.969	0.325	0.79
Sequencing vs. rsCDM using MEM at 4 h	2.099	0.147	0.83
Sequencing vs. rsCDM using MEM at 6 h	1.034	0.309	0.88
eCIM vs. rsCDM using MEM at 4 h	0.011	0.915	0.85
eCIM vs. rsCDM using MEM at 6 h	0.292	0.589	0.83

*IMI, imipenem; MEM, meropenem.*

## Discussion

Studies (Rodríguez-Baño et al., 2018; Durante-Mangoni et al., 2019; Tilahun et al., 2021) have noted that therapies varied from different β-lactamases of CRE. Hence, detecting and distinguishing carbapenemases is critical for selecting therapies. Accordingly, various phenotypic methods (Kalantar-Neyestanaki and Fatahi Bafghi, 2015; Jing et al., 2018, 2019; Tamma et al., 2019; CLSI, 2020) can be performed in most routine laboratories, of which lateral flow immunoassays and commercial MALDI TOF MS are unsuitable due to higher cost ($2–$10 per test) compared to rsCDM (less than $1 per test) (Tamma and Simner, 2018). eCIM and mCIMplus methods are low cost (less than $1 per test) but need 16–20 h to classify carbapenemase. However, rsCDM could provide reliable results within 4 h for classification of carbapenemases (KPC, NDM, VIM, IMP, KPC, and NDM) and AmpC enzymes with abnormalities in *blaOmpK35/blaOmpK36*, which is more beneficial for therapeutic decision and infection control. Furthermore, rsCDM exhibited a high sensitivity in the detection of CPE compared with the original method (Giske et al., 2011; Silva et al., 2017). In the actual test, even if technicians were unable to read the inhibition zones in 4–6 hours, the results remained unchanged over time, and the 18 h result was consistent with the 6 h result.

As presented in Table 1, rsCDM showed satisfactory performance in identifying carbapenemase at 4, 6, and 18 h. Notably, accurate characterization was obtained for 97.1% of tested CPE isolates and a categorical agreement between 98.6 and 100% depending on the enzyme class, based on the fact that MBLs, class A carbapenemases (KPC) and AmpC can be inhibited by EDTA, APBA, APBA, and CLO, respectively (Giske et al., 2011; Silva et al., 2017). Six AmpC-producing *Klebsiella pneumoniae* strains were unclassified at 4 h. After 6 h, all of them were positive for imipenem and two for meropenem. Since the MIC of ertapenem was ≥ 8 mg/L, six AmpC-producing strains were rechecked by ertapenem. The unsatisfactory results could be because ertapenem was less stable than imipenem, a potent inducer of AmpC hyper-production and it remained stable against hydrolysis by forming an acyl enzyme complex (Sanders et al., 1997; Tamma et al., 2019). Similar to ertapenem, meropenem was more vulnerable to hydrolysis of AmpC than imipenem. Therefore, for suspected AmpC-producing CRE strains, imipenem is a better choice than meropenem and ertapenem for verifying the phenotype, while for common KPC, NDM and IMP carbapenemases, both imipenem and meropenem can be used for detecting.

No positive results were obtained with five OXA-181-producing strains and three ESBL-producing *K. pneumoniae* isolates with porin changes, since OXA-181 and ESBL enzyme cannot be inhibited by EDTA, APBA, and CLO, which is the disadvantage of most phenotypic assays (Silva et al., 2017; Jing et al., 2018, 2019; Liao et al., 2019, 2021). The Ambler classification of CRE cannot be interpreted by the algorithm, and the tested strains may produce AmpC/ESBL associated with decreased permeability and/or production of OXA-48-like enzymes. Meropenem MICs of three ESBL-producing *K. pneumoniae* were 4, 4, 1 mg/L, imipenem 2, 2, 1 mg/L, and ertapenem ≥ 8, 8, 4 mg/L, respectively, which were consistent with the results obtained by Birgy et al. (2012) and Tamma et al. (2017), ertapenem had higher MICs than meropenem and imipenem for ESBL/AmpC-producing CRE isolates. Thus, the efficacy of ertapenem was mainly impaired by decreased permeability (Birgy et al., 2012). The isolate showed intermediate or low-level resistance to imipenem and meropenem but high-level resistance to ertapenem (MIC ≥ 8 mg/L), which suggests that the mechanism of CRE was ESBL or AmpC along with porin changes.

In a previous study (Liao et al., 2021), using meropenem disk to detect 0.5 McFarland IMP-isolates could be interpreted more easily than imipenem since the former had a stronger bacteriostatic effect (Guzek et al., 2013). However, no similar phenomenon was observed when the bacterial concentration increased to 3.0 McFarland. In addition, CPE isolates with mucoid characteristic were false negative by phenotypic assays in previous studies (Liao et al., 2019, 2021), but not in this study. In the disk diffusion procedure, the growth of bacteria is inhibited at a certain concentration of antibiotic, and when higher concentrations of bacteria are inoculated, the bacterial colonies become visible in shorter incubation durations, so the rsCDM results could be interpreted at 4 h. The reason for not choosing a higher concentration of suspension was that the upper limit of the DENSIMAT is 3.0 McFarland. Since complete inhibition needs a certain incubation period, some strains will develop a thin biofilm at 4 h, which requires measuring the zone diameter against light, ignoring moss growth. As the culture duration increases to 6 h, the film-like growth significantly decreases and interpretation of the results become easier, which could be reflected by the increase in the subtraction diameter of the inhibitory zones.

This study had several limitations. Given the local epidemiological conditions, we only tested the major carbapenemase types and a limited number of non-CP-CRE isolates. Further studies with more isolates, notably VIM, IMP, OXA-48 like, double carbapenemase, and AmpC in combination with *blaOmpK35/blaOmpK36* changes producers, are required to confirm the performance of this test. Furthermore, strains carrying other class A carbapenemases such as IMI and GES were not included in this study since they are rarely isolated in China (Wang et al., 2018).

## Conclusion

The rsCDM offers several advantages in diagnostic performance characteristics, labor intensity, cost, and turnaround time (TAT), which permits therapeutic decision-making and infection control in a shorter time. It is easy to perform, simple to interpret, accessible and accurate technique requiring only basic laboratory equipment.

## Data Availability Statement

The original contributions presented in the study are included in the article/Supplementary Material, further inquiries can be directed to the corresponding author/s.

## Author Contributions

MK, QL, and YY: conception and design of the study. QL and YY: analysis and interpretation of data and statistical analysis. QL, YY, WZ, JD, SW, YL, and YX: methodology. QL: drafting the manuscript. MK: revision of manuscript. All authors: final approval of manuscript.

## Conflict of Interest

The authors declare that the research was conducted in the absence of any commercial or financial relationships that could be construed as a potential conflict of interest.

## Publisher’s Note

All claims expressed in this article are solely those of the authors and do not necessarily represent those of their affiliated organizations, or those of the publisher, the editors and the reviewers. Any product that may be evaluated in this article, or claim that may be made by its manufacturer, is not guaranteed or endorsed by the publisher.
