# Supplementary material for: Detection and Characterization of Carbapenemases in Enterobacterales With a New Rapid and Simplified Carbapenemase Detection Method Called rsCDM
Source: Front Microbiol. 2022 Apr 28;13:860288. doi: 10.3389/fmicb.2022.860288 (PMC9097014; doi:10.3389/fmicb.2022.860288)
Supplement: Supplementary file 1 [file Table_1.docx]

Table S1: MICs (mg/L) for carbapenems, rsCDM and eCIM results for all isolates

| Isolate | Species | Carbapenemase(s) | MICs (mg/L) | | | rsCDM with IMI at 4 h | rsCDM with IMI at 6 h | rsCDM with MEM at 4 h | rsCDM with MEM at 6 h | eCIM |
| --- | --- | --- | --- | --- | --- | --- | --- | --- | --- | --- |
|  |  |  | MEM | IMI | ERT |  |  |  |  |  |
| Carbapenemase-producing *Enterobacterales* (n=173) | | | | | | | | | | |
| 124R | *K. pneumoniae* | KPC-2 | ≥16 | 8 | ≥8 | + | + | + | + | -* |
| 128R | *K. pneumoniae* | KPC-2 | ≥16 | ≥16 | ≥8 | + | + | + | + | -* |
| 129R | *K. pneumoniae* | KPC-2 | ≥16 | ≥16 | ≥8 | + | + | + | + | -* |
| 130R | *K. pneumoniae* | KPC-2 | ≥16 | ≥16 | ≥8 | + | + | + | + | -* |
| 131R | *K. pneumoniae* | KPC-2 | ≥16 | ≥16 | ≥8 | + | + | + | + | -* |
| 132R | *K. pneumoniae* | KPC-2 | ≥16 | ≥16 | ≥8 | + | + | + | + | -* |
| 134R | *K. pneumoniae* | KPC-2 | ≥16 | ≥16 | ≥8 | + | + | + | + | -* |
| 135R | *K. pneumoniae* | KPC-2 | ≥16 | ≥16 | ≥8 | + | + | + | + | -* |
| 136R | *K. pneumoniae* | KPC-2 | 32 | 32 | ≥8 | + | + | + | + | -* |
| 201R | *K. pneumoniae* | KPC-2 | ≥16 | ≥16 | ≥8 | + | + | + | + | -* |
| 202R | *K. pneumoniae* | KPC-2 | ≥16 | ≥16 | ≥8 | + | + | + | + | -* |
| 203R | *K. pneumoniae* | KPC-2 | ≥16 | ≥16 | ≥8 | + | + | + | + | -* |
| 204R | *K. pneumoniae* | KPC-2 | ≥16 | ≥16 | ≥8 | + | + | + | + | -* |
| 205R | *K. pneumoniae* | KPC-2 | ≥16 | ≥16 | ≥8 | + | + | + | + | -* |
| 206R | *K. pneumoniae* | KPC-2 | ≥16 | ≥16 | ≥8 | + | + | + | + | -* |
| 207R | *K. pneumoniae* | KPC-2 | ≥16 | ≥16 | ≥8 | + | + | + | + | -* |
| 208R | *K. pneumoniae* | KPC-2 | ≥16 | ≥16 | ≥8 | + | + | + | + | -* |
| 209R | *K. pneumoniae* | KPC-2 | ≥16 | ≥16 | ≥8 | + | + | + | + | -* |
| 210R | *K. pneumoniae* | KPC-2 | ≥16 | ≥16 | ≥8 | + | + | + | + | -* |
| 102R | *K. pneumoniae* | KPC-2 | ≥16 | ≥16 | ≥8 | + | + | + | + | -* |
| 104R | *K. pneumoniae* | KPC-2 | ≥16 | ≥16 | ≥8 | + | + | + | + | -* |
| 105R | *K. pneumoniae* | KPC-2 | ≥16 | ≥16 | ≥8 | + | + | + | + | -* |
| 110R | *K. pneumoniae* | KPC-2 | ≥16 | ≥16 | ≥8 | + | + | + | + | -* |
| 111R | *K. pneumoniae* | KPC-2 | ≥16 | ≥16 | ≥8 | + | + | + | + | -* |
| 114R | *K. pneumoniae* | KPC-2 | ≥16 | ≥16 | ≥8 | + | + | + | + | -* |
| 117R | *K. pneumoniae* | KPC-2 | ≥16 | ≥16 | ≥8 | + | + | + | + | -* |
| 118R | *K. pneumoniae* | KPC-2 | ≥16 | ≥16 | ≥8 | + | + | + | + | -* |
| 120R | *K. pneumoniae* | KPC-2 | ≥16 | ≥16 | ≥8 | + | + | + | + | -* |
| 121R | *K. pneumoniae* | KPC-2 | ≥16 | 8 | ≥8 | + | + | + | + | -* |
| 122R | *K. pneumoniae* | KPC-2 | ≥16 | ≥16 | ≥8 | + | + | + | + | -* |
| 123R | *K. pneumoniae* | KPC-2 | ≥16 | ≥16 | ≥8 | + | + | + | + | -* |
| 154R | *K. pneumoniae* | KPC-2 | ≥16 | ≥16 | ≥8 | + | + | + | + | -* |
| 15R | *K. pneumoniae* | KPC-2 | ≥16 | ≥16 | ≥8 | + | + | + | + | -* |
| 16R | *K. pneumoniae* | KPC-2 | ≥16 | ≥16 | ≥8 | + | + | + | + | -* |
| 17R | *K. pneumoniae* | KPC-2 | ≥16 | ≥16 | ≥8 | + | + | + | + | -* |
| 18R | *K. pneumoniae* | KPC-2 | ≥16 | ≥16 | ≥8 | + | + | + | + | -* |
| 21R | *K. pneumoniae* | KPC-2 | ≥16 | ≥16 | ≥8 | + | + | + | + | -* |
| 23R | *K. pneumoniae* | KPC-2 | ≥16 | ≥16 | ≥8 | + | + | + | + | -* |
| 26R | *K. pneumoniae* | KPC-2 | ≥16 | ≥16 | ≥8 | + | + | + | + | -* |
| 27R | *K. pneumoniae* | KPC-2 | ≥16 | ≥16 | ≥8 | + | + | + | + | -* |
| 28R | *K. pneumoniae* | KPC-2 | ≥16 | ≥16 | ≥8 | + | + | + | + | -* |
| 29R | *K. pneumoniae* | KPC-2 | ≥16 | ≥16 | ≥8 | + | + | + | + | -* |
| 30R | *K. pneumoniae* | KPC-2 | >32 | >32 | ≥8 | + | + | + | + | -* |
| 31R | *K. pneumoniae* | KPC-2 | ≥16 | ≥16 | ≥8 | + | + | + | + | -* |
| 33R | *K. pneumoniae* | KPC-2 | ≥16 | ≥16 | ≥8 | + | + | + | + | -* |
| 34R | *K. pneumoniae* | KPC-2 | ≥16 | ≥16 | ≥8 | + | + | + | + | -* |
| 36R | *K. pneumoniae* | KPC-2 | ≥16 | ≥16 | ≥8 | + | + | + | + | -* |
| 37R | *K. pneumoniae* | KPC-2 | >32 | >32 | ≥8 | + | + | + | + | -* |
| 38R | *K. pneumoniae* | KPC-2 | >32 | >32 | >32 |  | + | + | + | -* |
| 40R | *K. pneumoniae* | KPC-2 | >32 | >32 | ≥8 | + | + | + | + | -* |
| 45R | *K. pneumoniae* | KPC-2 | ≥16 | ≥16 | ≥8 | + | + | + | + | -* |
| 46R | *K. pneumoniae* | KPC-2 | ≥16 | ≥16 | ≥8 | + | + | + | + | -* |
| 47R | *K. pneumoniae* | KPC-2 | ≥16 | ≥16 | ≥8 | + | + | + | + | -* |
| 49R | *K. pneumoniae* | KPC-2 | ≥16 | ≥16 | ≥8 | + | + | + | + | -* |
| 50R | *K. pneumoniae* | KPC-2 | ≥16 | ≥16 | ≥8 | + | + | + | + | -* |
| 52R | *K. pneumoniae* | KPC-2 | ≥16 | ≥16 | ≥8 | + | + | + | + | -* |
| 53R | *K. pneumoniae* | KPC-2 | ≥16 | ≥16 | ≥8 | + | + | + | + | -* |
| 54R | *K. pneumoniae* | KPC-2 | ≥16 | ≥16 | ≥8 | + | + | + | + | -* |
| 55R | *K. pneumoniae* | KPC-2 | ≥16 | ≥16 | ≥8 | + | + | + | + | -* |
| 57R | *K. pneumoniae* | KPC-2 | ≥16 | ≥16 | ≥8 | + | + | + | + | -* |
| 58R | *K. pneumoniae* | KPC-2 | ≥16 | ≥16 | ≥8 | + | + | + | + | -* |
| 59R | *K. pneumoniae* | KPC-2 | ≥16 | ≥16 | ≥8 | + | + | + | + | -* |
| 60R | *K. pneumoniae* | KPC-2 | ≥16 | ≥16 | ≥8 | + | + | + | + | -* |
| 61R | *K. pneumoniae* | KPC-2 | ≥16 | ≥16 | ≥8 | + | + | + | + | -* |
| 62R | *K. pneumoniae* | KPC-2 | ≥16 | ≥16 | ≥8 | + | + | + | + | -* |
| 65R | *K. pneumoniae* | KPC-2 | >32 | >32 | ≥8 | + | + | + | + | -* |
| 67R | *K. pneumoniae* | KPC-2 | ≥16 | ≥16 | ≥8 | + | + | + | + | -* |
| 69R | *K. pneumoniae* | KPC-2 | ≥16 | ≥16 | ≥8 | + | + | + | + | -* |
| 71R | *K. pneumoniae* | KPC-2 | ≥16 | ≥16 | ≥8 | + | + | + | + | -* |
| 72R | *K. pneumoniae* | KPC-2 | ≥16 | ≥16 | ≥8 | + | + | + | + | -* |
| 74R | *K. pneumoniae* | KPC-2 | ≥16 | ≥16 | ≥8 | + | + | + | + | -* |
| 75R | *K. pneumoniae* | KPC-2 | ≥16 | ≥16 | ≥8 | + | + | + | + | -* |
| 76R | *K. pneumoniae* | KPC-2 | ≥16 | ≥16 | ≥8 | + | + | + | + | -* |
| 77R | *K. pneumoniae* | KPC-2 | ≥16 | ≥16 | ≥8 | + | + | + | + | -* |
| 78R | *K. pneumoniae* | KPC-2 | ≥16 | ≥16 | ≥8 | + | + | + | + | -* |
| 79R | *K. pneumoniae* | KPC-2 | ≥16 | ≥16 | ≥8 | + | + | + | + | -* |
| 80R | *K. pneumoniae* | KPC-2 | ≥16 | ≥16 | ≥8 | + | + | + | + | -* |
| 81R | *K. pneumoniae* | KPC-2 | ≥16 | ≥16 | ≥8 | + | + | + | + | -* |
| 82R | *K. pneumoniae* | KPC-2 | ≥16 | ≥16 | ≥8 | + | + | + | + | -* |
| 84R | *K. pneumoniae* | KPC-2 | ≥16 | ≥16 | ≥8 | + | + | + | + | -* |
| 86R | *K. pneumoniae* | KPC-2 | ≥16 | ≥16 | ≥8 | + | + | + | + | -* |
| 87R | *K. pneumoniae* | KPC-2 | ≥16 | ≥16 | ≥8 | + | + | + | + | -* |
| 88R | *K. pneumoniae* | KPC-2 | ≥16 | ≥16 | ≥8 | + | + | + | + | -* |
| 89R | *K. pneumoniae* | KPC-2 | ≥16 | ≥16 | ≥8 | + | + | + | + | -* |
| 90R | *K. pneumoniae* | KPC-2 | ≥16 | ≥16 | ≥8 | + | + | + | + | -* |
| 92R | *K. pneumoniae* | KPC-2 | ≥16 | ≥16 | ≥8 | + | + | + | + | -* |
| 95R | *K. pneumoniae* | KPC-2 | ≥16 | ≥16 | ≥8 | + | + | + | + | -* |
| 090570 | *E. coli* | *OXA-181* | >32 | >32 | ≥8 | - | - | - | - | -* |
| 115057 | *K. pneumoniae* | *OXA-181* | 2 | 1.5 | ≥8 | - | - | - | - | -* |
| 140071 | *K. pneumoniae* | *OXA-181* | 1 | 1 | 4 | - | - | - | - | -* |
| 005828 | *E. coli* | *OXA-181* | >32 | >32 | ≥8 | - | - | - | - | -* |
| RJJ | *K. pneumoniae* | *OXA-181* | ≥16 | ≥16 | ≥8 | - | - | - | - | -* |
| 98R | *K. pneumoniae* | KPC-2 | ≥16 | ≥16 | ≥8 | + | + | - | + | -* |
| 119R | *K. pneumoniae* | NDM-5 | ≥16 | ≥16 | ≥8 | + | + | + | + | + |
| 112 R | *K. pneumoniae* | NDM-5 | ≥16 | ≥16 | ≥8 | + | + | + | + | + |
| 106 R | *K. pneumoniae* | NDM-5 | ≥16 | ≥16 | ≥8 | + | + | + | + | + |
| 63 R | *K. pneumoniae* | NDM-5 | ≥16 | ≥16 | ≥8 | + | + | + | + | + |
| 42R | *K. pneumoniae* | NDM-5 | ≥16 | ≥16 | ≥8 | + | + | + | + | + |
| 51R | *K. pneumoniae* | NDM-5 | ≥16 | ≥16 | ≥8 | + | + | + | + | + |
| 48R | *K. pneumoniae* | NDM-5 | ≥16 | ≥16 | ≥8 | + | + | + | + | + |
| 127R | *K. pneumoniae* | NDM-5 | ≥16 | ≥16 | ≥8 | + | + | + | + | + |
| 41R | *K. pneumoniae* | NDM-5 | 32 | 32 | ≥8 | + | + | + | + | + |
| 137R | *K. pneumoniae* | NDM-5 | ≥16 | ≥16 | ≥8 | + | + | + | + | + |
| 45653R | *Enterbacter hormaechei* | NDM-5 | >32 | >32 | ≥8 | + | + | + | + | + |
| 2001R | *E. coli* | NDM-5 | >32 | >32 | ≥8 | + | + | + | + | + |
| D7 | *E. coli* | NDM-5 | >32 | >32 | ≥8 | + | + | + | + | + |
| D4 | *E. coli* | NDM-5 | >32 | >32 | ≥8 | + | + | + | + | + |
| D1 | *E. coli* | NDM-5 | >32 | >32 | ≥8 | + | + | + | + | + |
| D6 | *Enterbacter hormaechei* | NDM-1 | 4 | 4 | ≥8 | + | + | + | + | + |
| D5 | *E. coli* | NDM-5 | >32 | >32 | ≥8 | + | + | + | + | + |
| D22 | *E. coli* | NDM-5 | >32 | >32 | ≥8 | + | + | + | + | + |
| D20 | *Citrobacter koseri* | NDM-1 | >32 | >32 | ≥8 | + | + | + | + | + |
| D16 | *E. coli* | NDM-5 | >32 | >32 | ≥8 | + | + | + | + | + |
| D24 | *Enterbacter hormaechei* | NDM-1 | >32 | 16 | ≥8 | + | + | + | + | + |
| D23 | *E. coli* | NDM-5 | >32 | >32 | ≥8 | + | + | + | + | + |
| D32 | *E. coli* | NDM-5 | >32 | >32 | ≥8 | + | + | + | + | + |
| D28 | *E. coli* | NDM-5 | >32 | >32 | ≥8 | + | + | + | + | + |
| D26 | *E. coli* | NDM-5 | >32 | >32 | ≥8 | + | + | + | + | + |
| D31 | *E. coli* | NDM-5 | >32 | >32 | ≥8 | + | + | + | + | + |
| 35R | *K. pneumoniae* | NDM-5 | >32 | >32 | ≥8 | + | + | + | + | + |
| 7R | *K. pneumoniae* | NDM-5 | >32 | >32 | ≥8 | + | + | + | + | + |
| 48R | *K. pneumoniae* | NDM-5 | ≥16 | ≥16 | ≥8 | + | + | + | + | + |
| 41R | *K. pneumoniae* | NDM-5 | 32 | 32 | ≥8 | + | + | + | + | + |
| D56 | *Enterbacter hormaechei* | NDM-1 | >32 | >32 | ≥8 | + | + | + | + | + |
| D43 | *Enterbacter hormaechei* | NDM-1 | >32 | >32 | ≥8 | + | + | + | + | + |
| D129 | *E. coli* | NDM-1 | >32 | >32 | ≥8 | + | + | + | + | + |
| D47 | *Enterbacter hormaechei* | NDM-1 | >32 | >32 | ≥8 | + | + | + | + | + |
| D44 | *E. coli* | NDM-5 | >32 | >32 | ≥8 | + | + | + | + | + |
| DJ85 | *E. coli* | NDM-5 | >32 | >32 | ≥8 | + | + | + | + | + |
| D123 | *E. coli* | NDM-5 | >32 | >32 | ≥8 | + | + | + | + | + |
| DJ38 | *Enterbacter hormaechei* | NDM-5 | >32 | >32 | ≥8 | + | + | + | + | + |
| D122 | *Enterbacter hormaechei* | NDM-1 | >32 | >32 | ≥8 | + | + | + | + | + |
| D33 | *E. coli* | NDM-5 | >32 | >32 | ≥8 | + | + | + | + | + |
| DJ88 | *E. coli* | NDM-5 | >32 | >32 | ≥8 | + | + | + | + | + |
| D76 | *E. coli* | NDM-7 | >32 | >32 | ≥8 | + | + | + | + | + |
| W18 | *E. cloacae* | NDM-1 | >32 | >32 | ≥8 | + | + | + | + | + |
| E13 | *E. cloacae* | NDM-1 | >32 | >32 | ≥8 | + | + | + | + | + |
| D33 | *E. coli* | NDM-5 | >32 | >32 | ≥8 | + | + | + | + | + |
| DJ43 | *Enterbacter hormaechei* | NDM-1 | >32 | >32 | ≥8 | + | + | + | + | + |
| D48 | *E. coli* | NDM-5 | >32 | >32 | ≥8 | + | + | + | + | + |
| DJ56 | *Enterbacter hormaechei* | NDM-1 | >32 | >32 | ≥8 | + | + | + | + | + |
| 2W | *E. cloacae* | NDM-1 | >32 | >32 | ≥8 | + | + | + | + | + |
| D123 | *E. coli* | NDM-5 | >32 | >32 | ≥8 | + | + | + | + | + |
| D38 | *Enterbacter hormaechei* | NDM-5 | >32 | >32 | ≥8 | + | + | + | + | + |
| D88 | *E. coli* | NDM-5 | >32 | >32 | ≥8 | + | + | + | + | + |
| D85 | *E. coli* | NDM-5 | >32 | >32 | ≥8 | + | + | + | + | + |
| D29 | *Enterbacter hormaechei* | NDM-1 | >32 | >32 | ≥8 | + | + | + | + | + |
| 133R | *K. pneumoniae* | NDM-5 | ≥16 | ≥16 | ≥8 | + | + | + | + | + |
| W15 | *E. cloacae* | NDM-1 | >32 | >32 | ≥8 | + | + | + | + | + |
| 211R | *E. coli* | NDM-5 | ≥16 | ≥16 | ≥8 | + | + | + | + | + |
| 212R | *E. coli* | NDM-5 | ≥16 | ≥16 | ≥8 | + | + | + | + | + |
| 213R | *E. coli* | NDM-5 | ≥16 | ≥16 | ≥8 | + | + | + | + | + |
| 214R | *E. coli* | NDM-5 | ≥16 | ≥16 | ≥8 | + | + | + | + | + |
| D55 | *K. pneumoniae* | IMP-4 | >32 | >32 | ≥8 | + | + | + | + | + |
| D53 | *K. pneumoniae* | IMP-4 | >32 | 4 | ≥8 | + | + | + | + | + |
| D52 | *K. pneumoniae* | IMP-4 | >32 | 4 | ≥8 | + | + | + | + | - |
| D54 | *Enterbacter hormaechei* | IMP-1 | >32 | 8 | ≥8 | + | + | + | + | + |
| 20052 | *K. pneumoniae* | IMP-4 | 4 | 4 | ≥8 | + | + | + | + | + |
| 20053 | *K. pneumoniae* | IMP-4 | 4 | 4 | ≥8 | + | + | + | + | + |
| D78 | *K. pneumoniae* | IMP-4 | 8 | 3 | ≥8 | + | + | + | + | + |
| 20055 | *K. pneumoniae* | IMP-4 | >32 | >32 | ≥8 | + | + | + | + | + |
| 115073 | *K. pneumoniae* | IMP-8 | >32 | >32 | ≥8 | + | + | + | + | + |
| P8 | *E. cloacae* | IPM-1 | >32 | 8 | ≥8 | + | + | + | + | + |
| P7 | *E. coli* | VIM-1 | 4 | 4 | ≥8 | + | + | - | + | + |
| W17 | *E. cloacae* | VIM-1 | ≥16 | ≥16 | ≥8 | + | + | + | + | + |
| P6 | *K. pneumoniae* | VIM-1 | ≥16 | ≥16 | ≥8 | + | + | + | + | + |
| 99R | *K. pneumoniae* | KPC-2+NDM-1 | ≥16 | ≥16 | ≥8 | + | + | + | + | -* |
| 43R | *K. pneumoniae* | KPC-2+NDM-1 | ≥16 | ≥16 | ≥8 | + | + | + | + | -* |
| 56R | *K. pneumoniae* | KPC-2+NDM-5 | ≥16 | ≥16 | ≥8 | + | + | + | + | -* |
| 115038 | *K. pneumoniae* | KPC-2+NDM-5 | >32 | >32 | ≥8 | + | + | + | + | -* |
| 115039 | *K. pneumoniae* | KPC-2+NDM-5 | >32 | >32 | ≥8 | + | + | + | + | + |
| 141R | *K. pneumoniae* | KPC-2+NDM-1 | ≥16 | ≥16 | ≥8 | + | + | + | + | -* |
| 115062 | *K. pneumoniae* | KPC-2+NDM-1 | >32 | >32 | ≥8 | + | + | + | + | -* |
| Non-carbapenemase-producing *Enterobacterales* (n=52) | | | | | | | | | | |
| 3R | *K. pneumoniae* | AmpC | 1 | 4 | ≥8 | - | + | - | - | N |
| 5R | *K. pneumoniae* | AmpC | 1.5 | 32 | ≥8 | - | + | - | - | N |
| 4R | *K. pneumoniae* | AmpC | 1.5 | 32 | ≥8 | - | + | - | - | N |
| L5 | *K. pneumoniae* | AmpC | 2 | 32 | ≥8 | - | + | - | - | N |
| L4 | *K. pneumoniae* | AmpC | 4 | 4 | ≥8 | - | + | - | + | N |
| 70R | *K. pneumoniae* | AmpC | 4 | 8 | ≥8 | - | + | - | + | N |
| 32R | *K. pneumoniae* | ESBL | 4 | 2 | >=8 | - | - | - | - | N |
| 83R | *K. pneumoniae* | ESBL | 4 | 2 | >=8 | - | - | - | - | N |
| 103R | *K. pneumoniae* | ESBL | 1 | 1 | 4 | - | - | - | - | N |
| 70S | *K. pneumoniae* | - | ≤ 1 | 1 | 0.5 | - | - | - | - | N |
| 71S | *K. pneumoniae* | - | 0.25 | 0.5 | 1 | - | - | - | - | N |
| 72S | *K. pneumoniae* | - | ≤ 1 | 1 | 0.5 | - | - | - | - | N |
| 73S | *E. coli* | - | ≤ 1 | ≤ 1 | 0.5 | - | - | - | - | N |
| 74S | *K. pneumoniae* | - | ≤ 1 | ≤ 1 | 0.5 | - | - | - | - | N |
| 75S | *K. pneumoniae* | - | ≤ 1 | ≤ 1 | 0.5 | - | - | - | - | N |
| 76S | *K. pneumoniae* | - | ≤ 1 | ≤ 1 | 0.5 | - | - | - | - | N |
| 77S | *K. pneumoniae* | - | 0.25 | 0.25 | 0.5 | - | - | - | - | N |
| 78S | *K. pneumoniae* | - | 0.25 | 0.5 | 1 | - | - | - | - | N |
| 79S | *E. coli* | - | ≤ 1 | ≤ 1 | 0.5 | - | - | - | - | N |
| 80S | *K. pneumoniae* | - | ≤ 1 | ≤ 1 | 0.5 | - | - | - | - | N |
| 81S | *K. pneumoniae* | - | ≤ 1 | ≤ 1 | 0.5 | - | - | - | - | N |
| 82S | *K. pneumoniae* | - | ≤ 1 | ≤ 1 | 0.5 | - | - | - | - | N |
| 83S | *K. pneumoniae* | - | ≤ 1 | ≤ 1 | 0.5 | - | - | - | - | N |
| 84S | *K. pneumoniae* | - | ≤ 1 | ≤ 1 | 0.5 | - | - | - | - | N |
| 85S | *E. coli* | - | ≤ 1 | ≤ 1 | 0.5 | - | - | - | - | N |
| 86S | *E. coli* | - | ≤ 1 | ≤ 1 | 0.5 | - | - | - | - | N |
| 87S | *K. pneumoniae* | - | ≤ 1 | ≤ 1 | 0.5 | - | - | - | - | N |
| 88S | *K. pneumoniae* | - | ≤ 1 | ≤ 1 | 0.5 | - | - | - | - | N |
| 89S | *K. pneumoniae* | - | ≤ 1 | ≤ 1 | 0.5 | - | - | - | - | N |
| 90S | *K. pneumoniae* | - | ≤ 1 | ≤ 1 | 0.5 | - | - | - | - | N |
| 91S | *K. pneumoniae* | - | ≤ 1 | ≤ 1 | 0.5 | - | - | - | - | N |
| 92S | *K. pneumoniae* | - | 0.25 | 0.5 | 1 | - | - | - | - | N |
| 93S | *K. pneumoniae* | - | 0.25 | 0.5 | 1 | - | - | - | - | N |
| 94S | *K. pneumoniae* | - | 0.25 | 0.5 | 1 | - | - | - | - | N |
| 95S | *K. pneumoniae* | - | ≤ 1 | ≤ 1 | 0.5 | - | - | - | - | N |
| 96S | *K. pneumoniae* | - | 0.25 | 0.25 | 0.5 | - | - | - | - | N |
| 97S | *K. pneumoniae* | - | ≤ 1 | ≤ 1 | 0.5 | - | - | - | - | N |
| 98S | *K. pneumoniae* | - | 0.25 | 0.25 | 0.5 | - | - | - | - | N |
| 99S | *K. pneumoniae* | - | 1 | 1 | 0.25 | - | - | - | - | N |
| 100S | *E. coli* | - | 0.125 | 0.094 | 0.5 | - | - | - | - | N |
| 101S | *K. pneumoniae* | - | ≤ 1 | ≤ 1 | 0.5 | - | - | - | - | N |
| 120S | *K. pneumoniae* | - | ≤ 1 | ≤ 1 | 0.5 | - | - | - | - | N |
| 103S | *K. pneumoniae* | - | ≤ 1 | ≤ 1 | 0.5 | - | - | - | - | N |
| 104S | *E. coli* | - | ≤ 1 | ≤ 1 | 0.5 | - | - | - | - | N |
| 105S | *K. pneumoniae* | - | 1 | 1 | 0.25 | - | - | - | - | N |
| 106S | *E. coli* | - | 1 | 1 | 0.25 | - | - | - | - | N |
| 107S | *K. pneumoniae* | - | ≤ 1 | ≤ 1 | 0.5 | - | - | - | - | N |
| 108S | *K. pneumoniae* | - | ≤ 1 | ≤ 1 | 0.5 | - | - | - | - | N |
| 109S | *K. pneumoniae* | - | ≤ 1 | ≤ 1 | 0.5 | - | - | - | - | N |
| 110S | *K. pneumoniae* | - | ≤ 1 | ≤ 1 | 0.5 | - | - | - | - | N |
| 112S | *K. pneumoniae* | - | ≤ 1 | ≤ 1 | 0.5 | - | - | - | - | N |
| 113S | *E. coli* | - | 0.125 | 0.125 | 0.5 | - | - | - | - | N |

MIC: minimal inhibitory concentration; MEM: meropenem; IMI: imipenem; ERT: ertapenem.

+: detection of carbapenemase activity; -: absence of detection of carbapenemase activity.

-*: detection of serine carbapenemase; N: not applicable.
